# Electrodeposited Fe on Cu foam as advanced fenton reagent for catalytic mineralization of methyl orange

**DOI:** 10.3389/fchem.2022.977980

**Published:** 2022-09-15

**Authors:** Modestas Vainoris, Aliona Nicolenco, Natalia Tsyntsaru, Elizabeth Podlaha-Murphy, Francisco Alcaide, Henrikas Cesiulis

**Affiliations:** ^1^ Faculty of Chemistry and Geosciences, Vilnius University, Vilnius, Lithuania; ^2^ Institute of Applied Physics of ASM, Chisinau, Moldova; ^3^ Departament de Física, Universitat Autònoma de Barcelona, Bellaterra, Spain; ^4^ Department of Chemical and Biomolecular Engineering Clarkson University, Potsdam, NY, United States; ^5^ CIDETEC, Basque Research and Technology Alliance (BRTA), Donostia-San Sebastián, Spain

**Keywords:** heterogeneous fenton reaction, electrodeposition, Fe/Cu catalyst, methyl orange, enviromentally friendly

## Abstract

In many countries, the textile industry remains the major contributor to environmental pollution. Untreated textile dyes discharged into water negatively impact the performance of aquatic organisms and may cause a variety of serious problems to their predators. Effective wastewater treatment is a key to reducing environmental and human health risks. In this work, the Fe/Cu catalysts were used in heterogeneous Fenton’s reaction for the degradation of high concentrations of methyl orange (model azo dye) in aqueous solutions. For the first time, the catalysts were prepared onto commercial copper foams by potentiostatic electrodeposition of iron using an environmentally friendly electrolyte. The influence of electrodeposition conditions, H_2_O_2_ concentration, dye concentration and temperature on the model dye degradation was investigated. It was revealed that both the surface area and the catalyst loading play the major role in the effective dye degradation. The experimental results involving spectrophotometric measurements coupled with total carbon and nitrogen quantification suggest that a solution containing up to 100 mg/L of methyl orange can be successfully decolorized within 90 s at 50°C using porous Fe/Cu catalyst in the presence of hydrogen peroxide that largely surpasses the current state-of-the-art performance. Already within the first 10°min, ∼ 30% of total methyl orange concentration is fully mineralized. The described process represents a cost-efficient and environmentally friendly way to treat azo dyes in aqueous solutions.

## Introduction

The production volume in the textile industry is increasing each year, which generates huge amounts of effluent containing dyes and other pollutants. Textile wastewater must be treated to remove or decrease the concentration of pollutants to the acceptable levels before its reuse or discharging into the environment (Holkar et al., 2016). This is especially the case of widely used azo dyes which contain one or more nitrogen-to-nitrogen double bonds (-N=N-) (Li et al., 2015; Sha et al., 2016), and 60–70% of dye groups and are fairly stable (Ananthashankar et al., 2013). Over the years various physical (coagulation, adsorption, reverse osmosis), chemical (ozonation and chlorination), and anaerobic biodegradation techniques have been developed to remove organic pollutants, including the azo dyes (Vandevivere et al., 1998; Murali et al., 2013). Usually, these techniques require expensive equipment, and do not fully mineralize all the pollutants but rather convert them into other organic compounds. In addition, the cleaning process may cause undesirable effects, e.g., chlorine gas evolution or precipitates, when chlorination is used as a water remediation procedure (Dutta et al., 2001; Tak and Kumar, 2017).

In recent years, advanced oxidation processes (AOPs) as a way to mineralize various organic dyes in waste water, and due to their high efficiency AOPs have drawn considerable attention. Also, decolorization dyes based on Fenton processes are of great interest (Miklos et al., 2018). Generally, this process involves a generation of the most reactive oxidizing agent in water treatment, namely reactive hydroxyl radicals, which non-selectively and rapidly react with numerous species. Since hydroxyl radicals have a very short lifetime, they are only produced *in-situ* through different chemical reactions processes. Fe(II) is the most frequently used because it can activate H_2_O_2_ and produce hydroxyl radicals in water (Deng and Zhao, 2015). In the Fenton process, H_2_O_2_ reacts with Fe^2+^ to generate strong reactive species.

The reactive species produced are traditionally recognized as hydroxyl radicals, though other substances such as ferryl-ions (also products of this reaction) (Deng and Zhao, 2015). In addition to Fe^2+^ ions, the oxidizing solution may contain other metal ions, e.g., Cu^+^, Cr^3+^, Co^2+^, Ti^3+^, W^6+^, Mo^n+^, etc., that can catalyze similar (Fenton-like) reactions (Wang et al., 2016; Zhang et al., 2018; Séverin et al., 2020). Remarkably, bimetallic Fenton reagents demonstrate a higher catalytic efficiency compared to the homogeneous Fe(II)-based catalysts. The activity of the catalyst can be greatly enhanced by combining Cu and Fe, where Cu can strongly accelerate the reduction cycle of Fe^3+^ to Fe^2+^ (Fe^2+^ has higher reaction rate when breaking down hydrogen peroxide) through exposed metal active sites and increase the accumulation of •OH oxidants in the system (Rossi et al., 2014). Heterogeneous Fe/Cu catalysts demonstrated great activity at various concentrations of organic matter as well as stability with multiples uses (Rossi et al., 2014; Wang Y. et al., 2015; Zhang et al., 2017).

Sludge management is an important factor that greatly affects the total treatment costs. One of the ways to reduce the amount of generated sludge is to use heterogeneous Fenton instead of homogeneous reaction, because the heterogeneous reaction has lower activation energy than the homogenous one (Karthikeyan et al., 2011), and higher removal efficiency (Fontecha-Cámara et al., 2011; Zárate-Guzmán et al., 2019). The overview of main advantages and disadvantages of different Fenton processes were discussed in details in (Xu et al., 2020). Moreover, the efficiency of the Fenton process can be manipulated by applying photo degradation and using various iron oxidation states for active hydroxyl radical’s generation (Devi et al., 2010).

Surface texturization is an energy-efficient approach to achieve high degree of organics breakdown. Highly active catalysts can be prepared by immobilizing (or depositing) iron species into porous templates, e.g., zeolites, activated carbon, etc., or using various nanoparticles (Fontecha-Cámara et al., 2011; Wang J. et al., 2015; Litter, 2017). However, these templates could clump up in real wastewater forming larger clusters.

**FIGURE 1:**
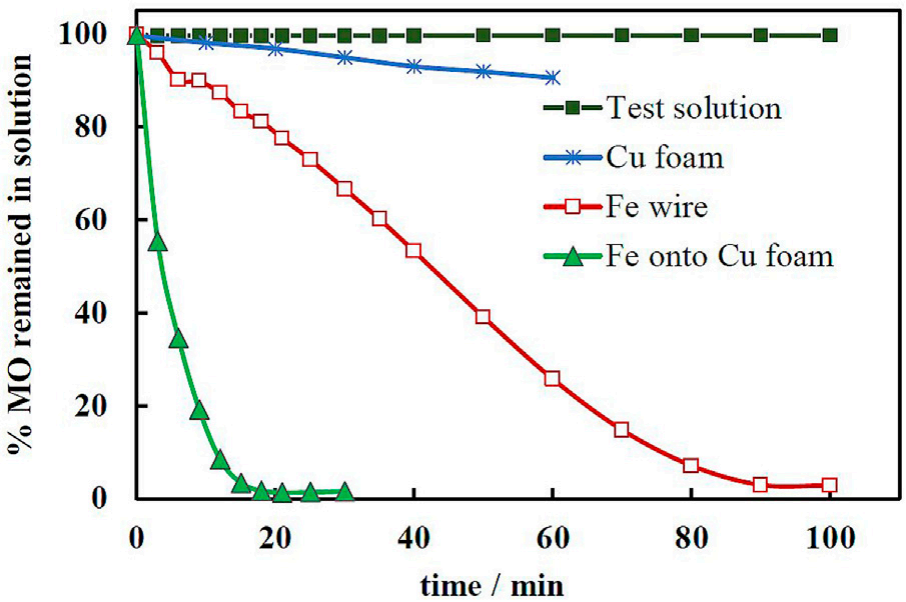
Preliminary evaluation of the catalytic activity of plane and 3D surface catalysts for degradation of MO. The geometrical area was 1 cm^2^ on all used substrates. The catalysts were electrodeposited at -1.5 V and q = 150 C. The test solution contained 100 mg/L of MO, 20 g/L Na_2_SO_4_ and 25 µL of H_2_O_2_.

Metal foam as a substrate for catalyst looks attractive because it has durability, ductility, light specific weight. In addition, such 3D porous materials have numerous applications in electrochemical technologies. Therefore, in this work, we fabricated an advanced Fe/Cu-foam catalyst for Fenton’s reaction, where a thin Fe coating was electrodeposited onto highly porous metallic copper foam. From the catalytic point of view, the importance has an “effective area” of catalyst in the reaction media, that in our case it is a solution. In order to estimate this parameter in the solution, the capacity of double electric layer (DEL) was chosen as an indicator of the effective surface area. DEL is thin enough in the moderately concentrated solutions (tens of nanometers), and replicates all surface irregularities that are higher than the thickness of DEL. The peculiarities of mass-transport, charge transfer and charging of DEL on the foam electrode were described in (Vainoris et al., 2020). The fabricated Fe/Cu-foam catalyst seems an attractive substrate for catalysts as the effective surface area of the foam is from 7 to 14 times bigger than the geometrical area of the smooth surface (Vainoris et al., 2020). Moreover, our preliminary results on the evaluation of the catalytic activity of plane surface (copper wire) vs. foam (Figure 1) confirm the effectiveness of the 3D substrate (taken into account that the geometrical area was the same). Therefore, in this paper we will be dealing with the foam substrate only.

In addition, the environmental issues were taken into account in this study, therefore for the electrodeposition process, i.e., an Fe(III)-based citrate-glycolate electrolyte (Mažeika et al., 2021) was used to ensure the stability of the bath over long periods of time and a high deposition rate. The activity of obtained catalysts was assessed using an aqueous solution of model nitro-dye, i.e., methyl orange (MO), investigating the rate of decolorization and degree of mineralization. The effects of various parameters that may influence the rate of Fenton-like heterogeneous reaction such as operating temperature, concentrations of dye and hydrogen peroxide were evaluated.

## Materials and methods

### Materials and sample preparation

All the chemicals were of analytical grade and were used without further purification (Carl Roth, Karlsruhe, Germany). Solutions have been prepared using deionized water (DI). Cu foam electrodes served as working electrodes were purchased from Alfa Aesar. The porosity of these electrodes is ∼90–91%. Electrochemical depositions have been performed and controlled using programmable potentiostat/galvanostat AUTOLAB PGSTAT 128N (Metrohm, Utrecht, Netherlands); the software used for controlling the hardware was Nova 1.11.2. All electrodepositions have been performed using a standard three-electrode cell, where Cu foam acted as a working electrode, circular platinized titanium mesh (Alfa Aesar, Ward Hill, MA, United States) was used as a counter electrode, and Ag/AgCl/KClsat (Sigma-Aldrich, St. Louis, MO, SA) was used as a reference electrode. All values of potentials are indicated in the text against this reference electrode. The distance between the counter and working electrode was fixed at 2.5 cm, and the distance between working and reference electrodes was fixed at 1.5 cm. The geometric size of working porous electrodes was 1 cm × 1 cm.

Prior measurements working electrodes have been washed and degreased using acetone, ethanol, and water in the ultrasonic bath. The native copper oxide layer has been removed by dipping copper foam into 2 M H_2_SO_4_ solution for 10 s and afterward rinsed with DI water. Electrodeposition of iron was carried out using a Fe(III)-based solution to avoid bath stability issues: 0.1 M Fe_2_(SO4)_3_, 0.3 M citric acid, and 1 M glycolic acid (Nicolenco et al., 2018). The pH of solutions was adjusted to 6.5 by sodium hydroxide and measured using a pH-meter ProLine Plus (Prosence B.V, Oosterhout, Netherlands). The electrodeposition was carried out at a constant temperature of 60°C (±0.5°C) and under constant stirring using a magnetic stirring bar at 600 rpm. Three distinct potentials have been chosen for electrodeposition: 1.5, −1.7, and −1.9 V with five corresponding amounts of charge for each potential applied: 150, 300, 450, 900, and 1350 C. All the depositions were replicated at least 8 times.

### Catalytic activity

MO aqueous solution was used to evaluate the catalytic activity of the modified foams. The test solution contained 40, 70, or 100 mg/L of MO and 20 g/L Na_2_SO_4_. The pH was adjusted to pH 3 (±0.1) using 95% H_2_SO_4_ solution. The temperature of the dye solution was kept at chosen value (30, 40, or 50°C) and constant stirring was applied using a magnetic stirring bar at 600 rpm. All the experiments were carried out in 100 ml cell. Hydrogen peroxide was added to the beaker with MO solution 1 min before the experiment, allowing the full distribution of it.

The modified copper foams were immersed into the dye solution for 10 min unless stated otherwise. The decolorization of the solution was tracked every minute by extracting 2 ml of solution and measuring the light absorption in 320–620 nm range (scan step 2 nm) using a spectrophotometer (T60 UV-Visible Spectrophotometer, PG Instruments Limited, United Kingdom). After the measurement, the aliquot was poured back into the beaker. The current efficiency was determined gravimetrically by tracking the weight of copper before deposition and after deposition of Fe.

The total organic content (TOC) and total nitrogen (TN) were determined using the TOC-VCSN Shimadzu analyzer (with TNM-1 block for nitrogen determination). The analysis was carried out after removing iron from the test solution by increasing pH to 9.8 using a 7 M NaOH solution. The formed iron oxides and hydroxides have been removed via centrifuge, and the supernatant was used for TOC and TN analysis.

### Characterization

The morphology of Fe-covered copper foams was investigated using a scanning electron microscope (SEM, Hitachi’s Tabletop Microscope TM-3000, Tokyo, Japan), equipped with an EDX module.

## Results and discussion

### Electrochemical iron deposition onto copper foam

Electrodeposition onto 3D substrates is generally challenging due to the complicated mass transport of charged species through the channels of the substrate. To obtain a good quality, the electrodeposition of Fe coating onto the Cu foam was performed under strict control of temperature and stirring. Three specific potential values were selected. For each one five distinct amounts of coulombs were let through the electrochemical cell. Figure 1 shows the surface morphology of obtained Fe/Cu catalysts.

**FIGURE 2:**
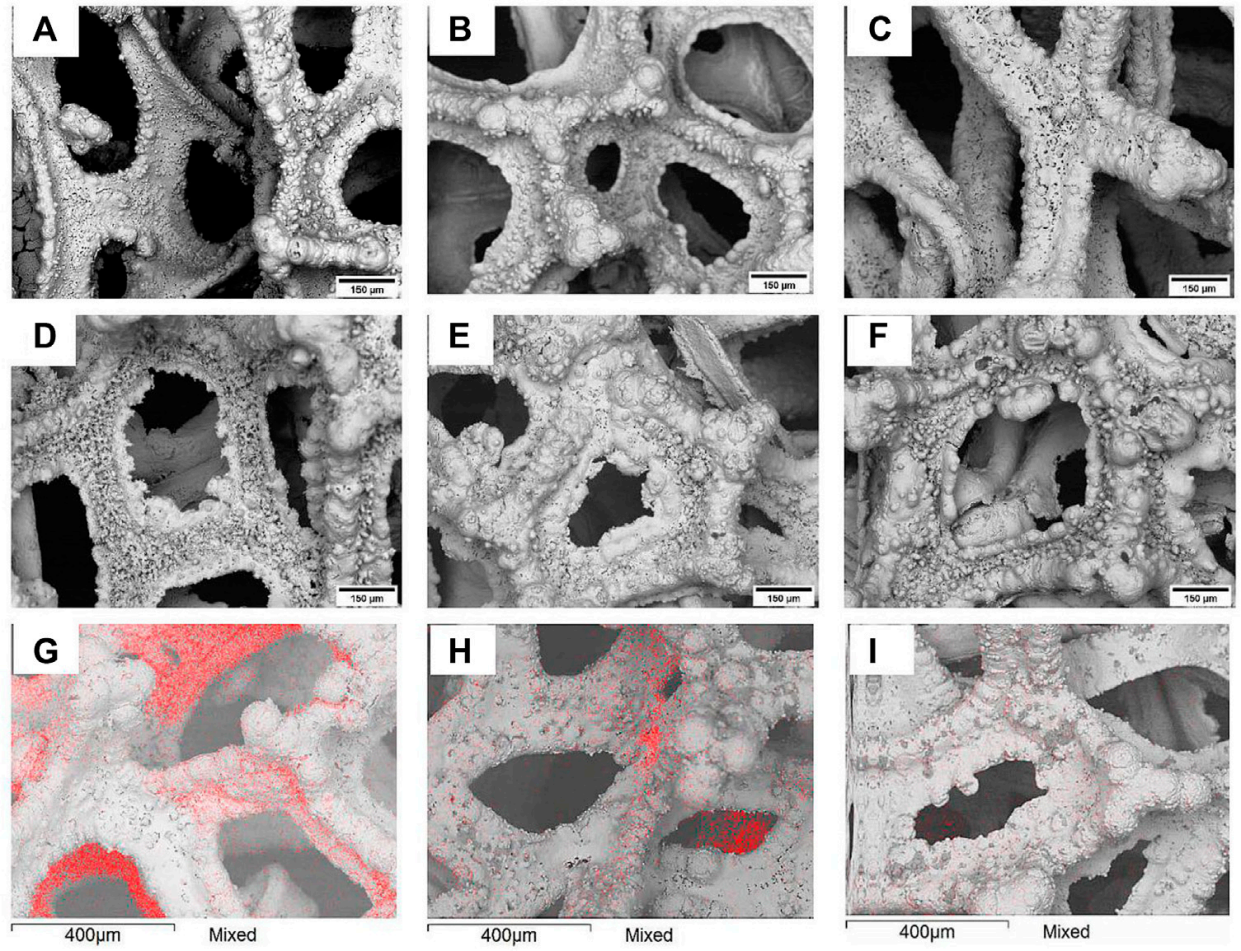
SEM images of electrochemically deposited copper under different conditions: top row q = 450 C **(A)** −1.5 V, **(B)** −1.7 V **(C)** −1.9 V; middle row—E = −1.9 V **(D)** q = 150 C, **(E)** q = 900 C, **(F)** q = 1350 C; bottom row—SEM/EDS map analysis, where the red color on the images indicates the Cu distribution at **(G)** 150 C, **(H)** 300 C, **(I)** 450 **(C)**.

With increasing the cathodic potential, Fe deposits become dendritic (see Figures 2A–C). It is worth mentioning that due to the complex shape of the copper foams all substrates exhibited a certain degree of edge effect, i.e., quicker growth and formation of an almost foam-like structure was observed at the edges of the foam substrate. Remarkably, such unevenly structured Fe deposits could provide more active sites for the Fenton reaction catalysis comparing to the flat surfaces. With increase in deposition time (i.e., charge passed, see Figures 2D–F), uneven bumps are developed on the surface, which could increase the surface area and enhance the activity of heterogeneous catalyst. Furthermore, the EDS map analysis (Figures 2G–I) reveals open copper parts at short electrodeposition time (60% of copper and 40% of iron), which are losing their visibility under prolonged time (increased charged passed).

**FIGURE 3:**
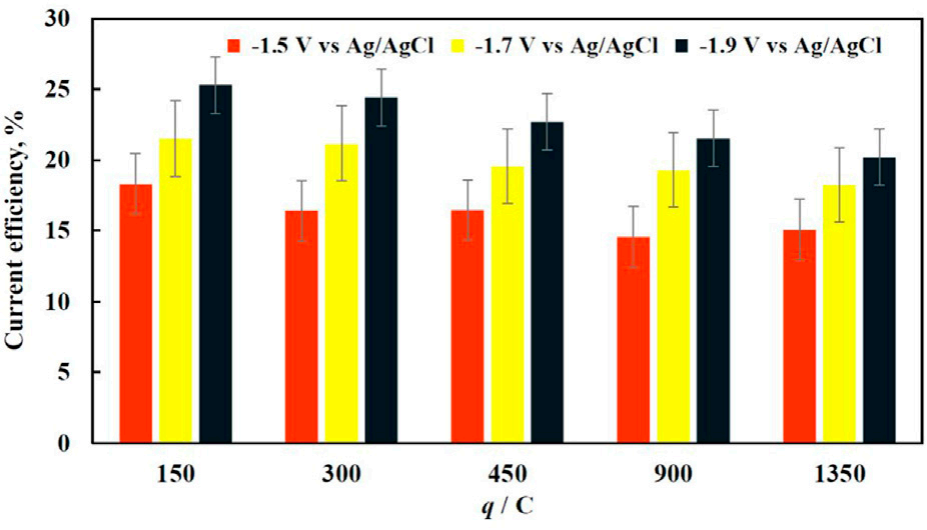
The dependence of current efficiency for iron deposition on the amount of charge passed through the electrochemical cell at various applied potentials (marked on the graph).

As it is seen in Figure 3, the current efficiency of the deposition did not exceed 30% regardless the potential applied. This is mainly caused by abundant hydrogen evolution reaction that accompanies iron electrodeposition and adsorption of organic matter on the working electrode that blocks the active surface of the electrode. At lower cathodic potentials, the current density is quite low, hence the rate of iron electrodeposition is quite slow and the surface coverage is not uniform probably due to electrocrystallization limits. With the increase in applied cathodic potential, there is a noticeable increase in the current efficiency, which is most likely caused by the interplay of partial current of side reactions and iron electrodeposition.

As the deposition time increases, the current efficiency drops regardless of the applied potential (Figure 3) probably due to the vigorous mixing that breaks apart the fragile dendrite-like structure. Nevertheless, the drop is relatively small, i.e., ∼3%. However, the decrease of current efficiency under longer deposition time becomes more significant at higher potentials. The morphology of iron deposits at −1.9 V vs. Ag/AgCl becomes foam-like (foam formation on the copper foam), and the moving electrolyte solution inside of the copper foams pores destroys this less mechanically stable structure. The drop in the current efficiency, when comparing the shortest and longest deposition times, becomes around 5% and is significant since the overall deposition efficiency reaches its maximum at approximately 25%.

### The effect of Fe electrodeposition potential on fenton reaction rate

To evaluate the effects of both the amount of deposited iron and eventual changes in morphology on the heterogenous Fenton reaction rate, the catalysts were electrochemically deposited under three distinct potentials with five different amounts of charge passed from 150 to 1300 C. The evaluation of catalytic activity was performed at 30°C tracking the average decolorization time in 70 mg/L MO solution in the presence of 90 µL of H_2_O_2_.

**FIGURE 4:**
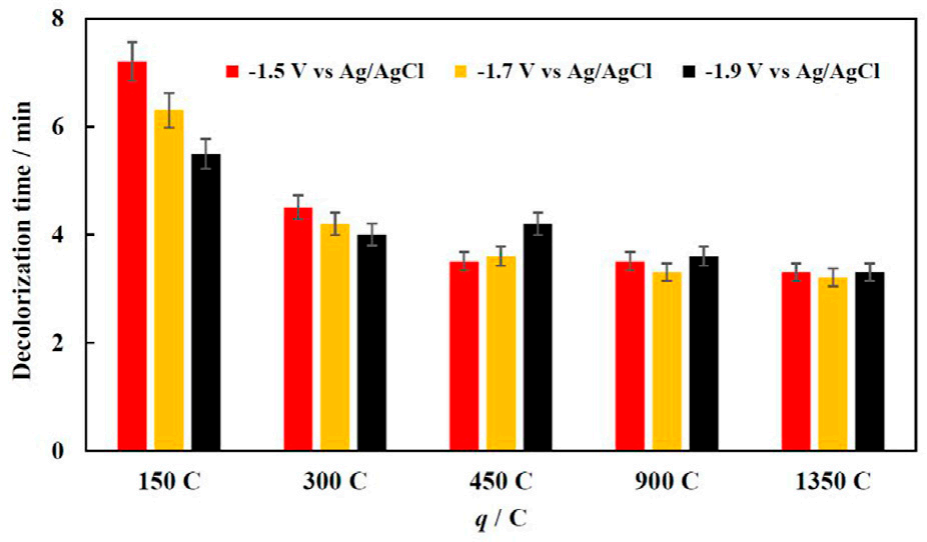
Effect of electrodeposition conditions (potential applied and charge passed) on decolorization time of 70 mg/L MO solution at 30°C.

The mechanism of MO degradation involves the cleavage of–N=N- bond with the formation of sulfanil ion and an aromatic amine and causes the disappearance of a characteristic color of the solution. The reaction process further through several steps that lead to full degradation of MO (Han et al., 2015). The obtained results are shown in Figure 4.

When 150 or 300 C are passed for Fe electrodeposition (thinner Fe film deposited and the smaller fraction of coverage Cu substrate by Fe), the decolorization time depends on both the potential and amount of passed charge. When Fe was deposited at higher potentials, the obtained Fe surface has more bumps and an almost foam-like structure (Figure 2), and in turn has more active sites. When a certain amount of iron is deposited on the copper foam, the Fenton reaction occurs at high enough rate, because the iron coating corrodes quickly in acidic media in the presence of H_2_O_2_ and forms enough Fe^2+^ ions, which later catalytically breakdown hydrogen peroxide into hydroxyl radicals, which in turn degrade organic dyes. The sequence of reactions occurs that within the modified Fenton process in the presence of Fe powder was proposed in (Gomathi Devi et al., 2009).

Further increasing the deposition time and amount of electrodeposited iron on the foam surface did not affect effect on the discoloration rate. The thick iron layer also prevents Cu to participate in a Fenton-like reaction, thus increasing the efficiency of the reaction by replenishing Fe^2+^ ions. However, increasing the amount of iron on the foam could reduce the mineralization efficiency, because of possible side reactions, which consume the ferrous ions and reduce the overall oxidation capacity.

### The effect of charge passed for Fe electrodeposition (*q*) at various MO concentrations

**FIGURE 5:**
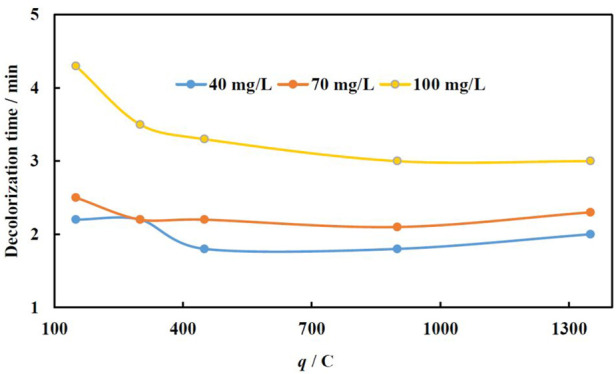
Dependence of decolorization time on MO concentration and catalyst deposition conditions (charge passed), at 40°C, 90 µL H_2_O_2_. Fe was electrodeposited at −1.9 V.

The effect of the amount of charge applied for Fe electrodeposition onto Cu foam on MO decolorization time was investigated at three concentrations of MO, i.e., 40, 70, and 100 mg/L, because these concentrations are close enough to those being found in the real wastewater (Yaseen and Scholz, 2019). The layers of Fe were electrodeposited at −1.9 V, because the best performance even at small *q* was obtained (for comparison see Figure 4). The other parameters were kept as 40°C and 90 μL of H_2_O_2_. The acquired dependences of decolorization time on charge amount passed for catalyst formation are shown in Figure 5.

The catalysts with a relatively low Fe load (less amount of *q* used for electroforming) and therefore higher fraction of exposed Cu substrate demonstrate a lower catalytic efficiency as compared to a catalyst with a higher load of Fe (higher amount of *q* used for electroforming). The same results were obtained for the catalysts with high Fe loading, i.e., produced at 1350 C, where the Cu substrate is fully covered, and no exposed regions are in contact with the solution.

### Effect of hydrogen peroxide concentration on fenton reaction rate

The mechanism of the Fenton reaction is quite complicated. Both heterogeneous and homogeneous reactions can occur simultaneously, and interdependencies exist between concentrations of organic matter, iron ions, and hydrogen peroxide. To determine the optimal H_2_O_2_ concentration, three concentrations of MO were chosen, and the catalyst with an excessive amount of iron (deposited at −1.9 V and *q* = 450 C) was used. The experiments were conducted at 30 and 40°C.

**FIGURE 6:**
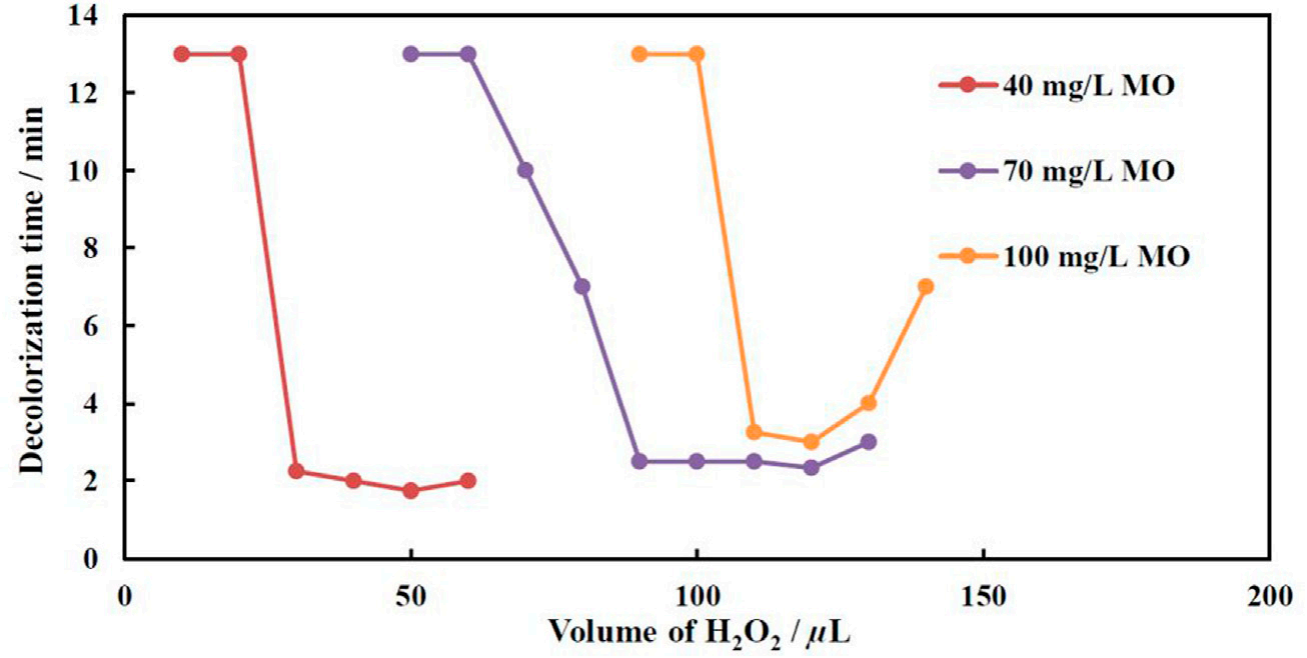
Effect of H_2_O_2_ concentration on decolorization time at various MO concentrations at 40°C. The catalyst was electrodeposited at −1.9 V, q = 450 C.

Figure 6 shows the variation of decolorization time as a function of the total H_2_O_2_ volume added to the MO solution. The fastest discoloration rate at lowest added H_2_O_2_ volumes was achieved for a solution containing the lowest concentration of MO, i.e., 40 mg/L. The results imply that the solution became transparent in less than 2 min after adding 50 µL of H_2_O_2_ (0.6 mm). Remarkably, when increasing MO concentration up to 100 mg/L, the optimal ratio between MO and hydrogen peroxide concentrations remains the same. Thus, for a 70 mg/L MO solution the optimal concentration of peroxide is 90 µL (1.1 mm), and for 100 mg/L MO—120 µL (or 1.2 mm of H_2_O_2_), that gives a factor of f = 0.8. This coefficient has been applied in all following experiments with variable concentration of MO in the solution. Note, that using a too low concentration of peroxide results either in a partial decolorization of the solution or a slower decolorization and only a partial mineralization. A large surplus of H_2_O_2_ does not increase the rate of discoloration. It is probably due to the complexity of the Fenton reaction (Gomathi Devi et al., 2009), where the initial concentration of organic compounds is directly responsible for decolorization time, and the rate-determining step of dye decolorization is the dye reaction with •OH radicals (Eq. 1):

$$•OH+Dye\to Oxidised\;dye+H_{2}O$$ (1)

It is surmised that under surplus of H_2_O_2_ the concentration of •OH radicals in the solution are kept high enough, and does not influence the decolorization rate of MO at relatively small concentrations of one (40–70 mg/L), whereas at a higher concentration of MO (100 mg/L) the consumption rate of •OH radicals became higher than the generation of one’s rate under reactions (Eqs. 2–5) (Gomathi Devi et al., 2009):

$${Fe}^{0}+H_{2}O_{2}\to {Fe}_{ads}^{2+}$$ (2)

$${Fe}_{ads}^{2+}+H_{2}O_{2}\to {Fe}^{3+}+2•OH$$ (3)

$${Fe}^{0}+H_{2}O_{2}\to {Fe}^{2+}+2OH^{-}$$ (4)

$${Fe}^{2+}+H_{2}O_{2}\to {Fe}^{3+}+•OH+{OH}^{-}$$ (5)

In addition, under excess of H_2_O_2_, the occurring passivation of iron results in the formation of thin passive layers (Namkung et al., 2005). Therefore, the reactions (2–4) are impeded, and the overall rate of dye oxidation reaction 1) decreases, i.e. decolorization time increases.

### Temperature effects on mineralization efficiency

**FIGURE 7:**
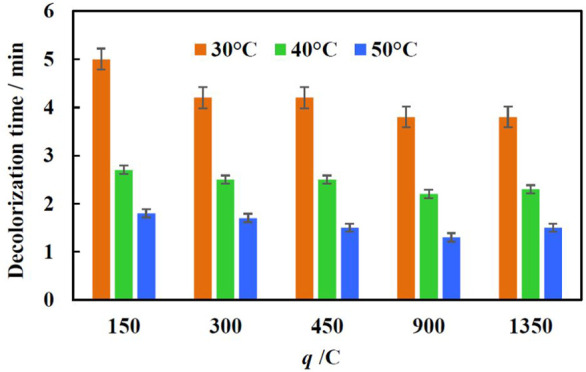
Dependence of decolorization time of 70 mg/L of MO solution (H_2_O_2_ conc.—90 µL) on temperature and amount of electric charge applied for iron deposition conditions.

Usually, the temperature of effluents from the textile wet processing is in the range of 30–60°C (Yaseen and Scholz, 2019). In order to simulate real effluents and investigate the effect of temperature on the degradations rate of MO, the catalysts were tested at three temperatures: 30, 40, and 50°C. Decolorization time was tracked at chosen temperatures in the 70 mg/L MO solution, using Fe/Cu catalyst electrodeposited at −1.9 V and 90 µL of H_2_O_2_. Figure 7 shows that the decolorization time difference between 30° and 50°C is almost 3 min and it is almost independent of the amount of iron on the copper foam. At 30°C the maximum efficiency is reached using catalysts obtained when 450 C and −1.9 V was applied for Fe layer electrodeposition. With further increase in temperature to 40 and 50°C, even a lower amount of iron on the copper foam is enough to reach minimal values of decolorization time, and there is no big change with the increase of charge passed. This further proves that under such conditions the overall reaction rate is being controlled by the iron corrosion rate. Since with the increase in temperature the corrosion rate increases as well. This implies that homogeneous Fenton’s reaction may occur in the bulk of the solution.

**FIGURE 8:**
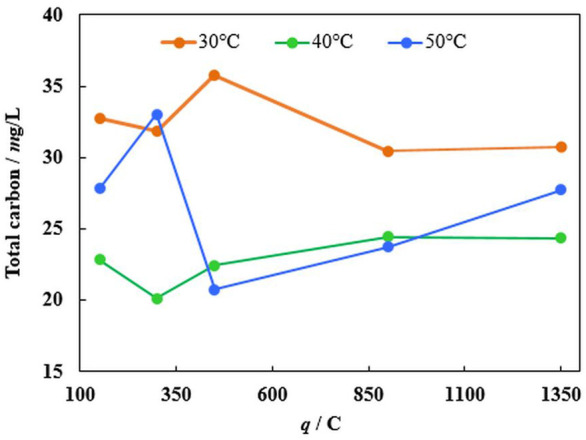
The dependence of total carbon amount after 10 min of degradation with immersed catalyst (obtained at −1.9 V) at various temperatures and iron loadings (charge passed).

The mineralization efficiency has been investigated to further evaluate the efficiency of the catalyst. The total amount of carbon in the solution has been determined by using catalysed oxidation of all organic matter at high temperatures. From Figure 8, the total MO degradation rate into carbon dioxide and water does not follow the same tendencies as decolorization time dependence on the temperature. Even though with increasing temperature the first degradation step, namely decolorization, occurs faster and the side reactions rates increase as well. However, the iron corrosion rate changes with the temperature, which likely controls the overall MO degradation rate.

**FIGURE 9:**
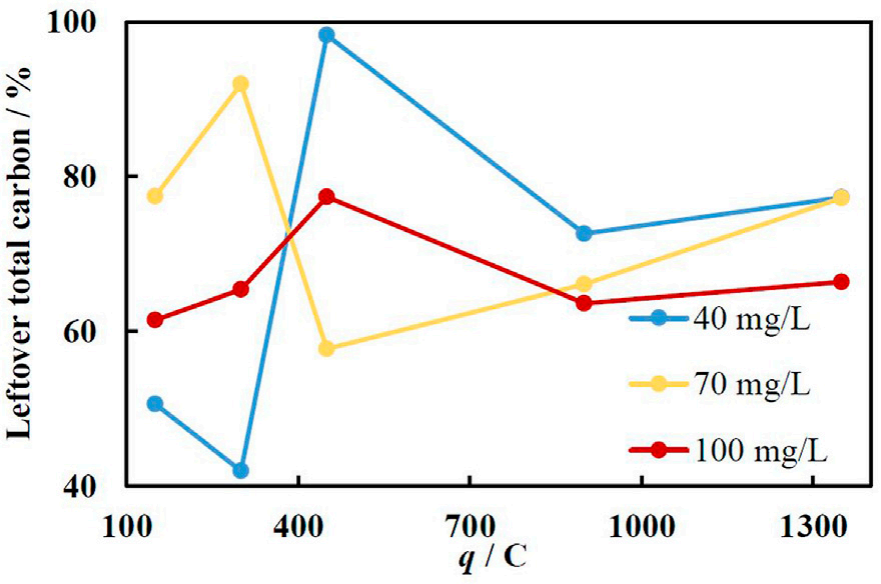
The dependence of total carbon amount in the solution on the current charge passed for Fe deposition (at −1.9 V) at various initial MO concentrations at 50°C. Immersion time 10 min.

Even though working at elevated temperatures may not be desirable, in practice this allows to digest higher concentration of pollutants. The results presented in Figure 9 demonstrate that only after 10 min at 50°C up to 50% of all organic matter was fully mineralized using Fe/Cu catalysts obtained at 150 and 300 C. At higher amounts of deposited iron, the leaching rate is too quick, and the H_2_O_2_ is likely used for water splitting and other side reactions. In the solution containing 70 mg/L of MO, the mineralization was quite weak at low catalysts loading, but from 450 C and upwards was around 25–35%. This is likely because the amount of iron on the Cu foam was not sufficient to achieve the reaction at its highest rate when the Fe loading was relatively low. The best results were obtained using 100 mg/L concentrations of MO, where the mineralization achieved in 10 min was around 25–40% and almost did not depend on the catalyst loading. This implies, that the iron ions were leached almost at the optimal rate. It is also worth mentioning that after all Fe layer is leached to the solution, the Cu foam substrate can be reused by electrodepositing a fresh portion of the catalyst.

Furthermore, various heterogeneous Fenton catalysts were compared, and the results are shown in Table 1. It can be noted that the effectiveness of catalysts is greatly dependent on the experimental conditions used, particularly the catalyst loading, dye, and H_2_O_2_ concertation. The Fe/Cu catalyst prepared in this work shows a relatively high efficiency compared with hollow Co. nanoparticles (Sha et al., 2016). With simple synthesis and ease of use, this catalyst shows great promise for upscaling and using it on an industrial scale.

**TABLE 1:** Comparison of various Fenton catalysts for the degradation of MO.

Type of catalyst	Catalyst amount	Solution volume (ml)	MO conc	Temperature	H_2_O_2_ conc	pH	Decolorization time	Ref
Ultrasonically dispersed nano metallic particles	50 mg/L	100	20 mg/L	-	50 mM	2.5	5 min	Singh et al. (2017)
Iron powder dispersed in solution along with UV illumination	10 mg	200	10 ppm		10 ppm	3.0	16 min	Gomathi Devi et al. (2009)
Homogeneous Fenton reaction	Fe^2+^ 0.2 mm		540 µm	Room temperature	3 mM	2.9	15 min	Youssef et al. (2016)
Hollow Co. nanoparticles	0.5 g/L	10	100 mg/L	Room temperature		2.5	4min	Sha et al. (2016)
Electro-Fenton-like process	2.14 mM Fe (II) I—2.1 A	500	106 mg/L			3.0	30 min	Taylor et al. (2013)
Zeolites modified with Fe-Mn/MCM41	1 g/L		100 mg/L	Room temperature	5 mmol/L	3.0	120 min	Zhang et al. (2018)
Tungsten oxide nanoparticles	50 mg	100		65 °C	2.5 ml	3.0	89.7% in 3h	Séverin et al. (2020)
Cu/Fe nanoribbons	14.5 mg		50 mg/L	Room temperature	0.1 g/L	3.0	20 min	Zhang et al. (2017)
Fe/Cu foam		100	100 mg/L	50°C	1.2 mm	3.0	1.5 min	This work

## Conclusion

The effective heterogeneous catalytic system containing Fe^0^ electrodeposited onto Cu foam was investigated in the Fenton process involving the methyl orange (MO) degradation. The influence of iron deposition conditions, H_2_O_2_ concentration and temperature on catalytic degradation of MO were investigated using various concentrations of MO in the solution. The optimal conditions for Fe layers deposition on Cu foam which provide the catalyst with a highest activity in Fenton process are: deposition potential −1.9 V vs Ag/AgCl at amount of charge >300 C and the geometric size of electrode 1 cm × 1 cm. This procedure allowed to achieve a full decolorization at a concentration of MO of 100 mg/L in up to 90 s depending on the temperature.

Moreover, the total mineralization efficiency is cca 30–40% already within the first 10 min after the catalyst immersion into the MO dye solution. The described catalytic process allows to effectively mineralize large amounts of MO within several minutes and represents an environmentally friendly and cost-effective alternative to other Fenton catalysts described in the literature.

## Data Availability

The raw data supporting the conclusions of this article will be made available by the authors, without undue reservation.
